# Clinical case study on custom 3D printed collars for dropped head syndrome patients

**DOI:** 10.1186/s41205-025-00274-x

**Published:** 2025-06-05

**Authors:** Abir Dutta, Jim Ashworth-Beaumont, Sanganagouda Patil, Kia Rezajooi, Deepak M Kalaskar

**Affiliations:** 1 Institute of Ortho and MSK Science, Division of Surgery and Interventional Sciences, University College London, London, NW3 2PF UK; 2 Department of Mechanical Engineering, Indian Institute of Technology Tirupati, Tirupati, 517619 Andhra Pradesh India; 3 Department of Orthotics, Royal National Orthopaedic Hospital-NHS Trust, Stanmore, HA7 4LP UK; 4 Spinal Surgery Unit, Royal National Orthopaedic Hospital-NHS Trust, Stanmore, HA7 4LP UK

**Keywords:** Cervical orthotic device, Neck collar, Dropped head syndrome, 3D printing, Case study

## Abstract

**Background:**

Dropped Head Syndrome (DHS) is a neurological condition characterized by severe head and neck muscle atrophy, leading to difficulties in maintaining a straight gaze and experiencing severe neck pain during daily activities. Standard off-the-shelf cervical orthotic devices (Neck Collars) often fail to provide adequate support for patients with DHS. This feasibility study aimed to develop and implement a novel feedback-incorporated workflow for creating personalized 3D printed (Powder Bed Fusion) cervical orthotic devices for six DHS patients with varying pathologies.

**Case presentation:**

A tailored workflow was devised and executed to produce bespoke 3D printed cervical orthotic devices for 6 DHS patients. The effectiveness of the collars in supporting patients during activities and reducing neck pain was assessed quantitatively and qualitatively using validated patient support questionnaires, Neck Disability Index, Visual Analog Score for Neck Pain, Global Cervical Angles (GCA), and Vertical Chin Brow Angles (VCBA) before and after intervention. Various clinical and design parameters were analysed to evaluate the collars’ efficacy in supporting patients and reducing neck pain. Patients exhibited an increase in GCA and a decrease in VCBA when using the collars as compared to their previous condition without those. The Visual Analog Score for Neck Pain decreased over the 6-month follow-up period, indicating positive implementation of the bespoke collars.

**Conclusion:**

The personalized design and functionality of the 3D printed collars significantly improved patients’ quality of life, representing a significant advancement in rehabilitative and supportive healthcare interventions. This pilot study lays the groundwork for further large-scale cohort studies.

**Supplementary Information:**

The online version contains supplementary material available at 10.1186/s41205-025-00274-x.

## Introduction

Dropped Head Syndrome (DHS) [1] is a challenging neurological disorder characterized by significant muscle atrophy in the head and neck region, resulting in weakness in the cervical and upper thoracic spine. This condition leads to severe pain and difficulties maintaining a straight gaze during everyday activities. Although DHS is not the sole cause of the dropped-head phenomenon, recent cases in the UK and EU have shown profound impacts on patients’ lifestyle [2–5].

Current treatment approaches primarily involve surgical intervention and medications. However, both before and after surgery, patients experience disruptions to their daily routines. Traditional cervical orthotic devices, commonly referred to as Neck Collars, offer limited support due to their one-size-fits-all design, failing to adequately assist patients in maintaining a straight head posture [6].

Early indications of customized cervical orthotic devices were documented by Hartman et al. in 1976, involving a 4-year-old boy [7]. Significant improvements were observed within a week of use. However, common issues such as patient tolerance and skin-related problems were noted with traditional cervical orthotic collars [8]. The importance of precise orthotist prescriptions was underscored.

Another instance involved in the design of a multi-segmented cervical orthotic device for a 19-year-old female with congenital torticollis. However, challenges persisted due to the complexity of the device and the time required for diagnosis to delivery. In 1998, Robert et al. reported on a newer cervical-thoracic orthotic device, but it lacked adequate support around the traps and neck due to its dependence on the user’s torso and waist measurements [9]. Although it showed potential for improving muscle weakness, emphasis was primarily on kyphosis patients, with limited evidence on patient comfort and clinical outcomes.

Subsequent innovations included a design tailored for DHS patients [10]^,^ limited to those with sufficient cervical extension range. Additionally, Reed et al. introduced a clothing-structure-based orthotic device in 2015, targeting motor neuron disease patients exclusively [11]. The unveiling of the ‘Headmaster Collar’ in 2019 marked progress, offering improved patient mobility. However, concerns arose regarding its lack of support for the back of the head and neck, limiting its applicability for all DHS patients [12]. Despite these advancements, long-term assessments and comprehensive patient feedback remained scarce, impeding efforts to refine and understand their effectiveness.

To date, reports [5, 13–16] on newer cervical orthotic devices and device-based surveys lack long-term assessments and patient feedback. Most studies focus on commercially available off-the-shelf collars, which often fail to provide an optimal fit due to varying patient morphologies. Notably, there is a dearth of literature on bespoke 3D printed cervical orthotic devices tailored specifically for DHS patients, indicating a significant research gap.

In this present study, with prescriptions from the spinal surgeons and advisory support from the orthotics team from collaborative hospital (name blinded), the workflow was adopted and modified based on previous study [17] (Fig. 1) and implemented to provide bespoke 3D printed (using Powder Bed Fusion) cervical orthotic devices for DHS patients.

## Methodology

**Fig. 1:**
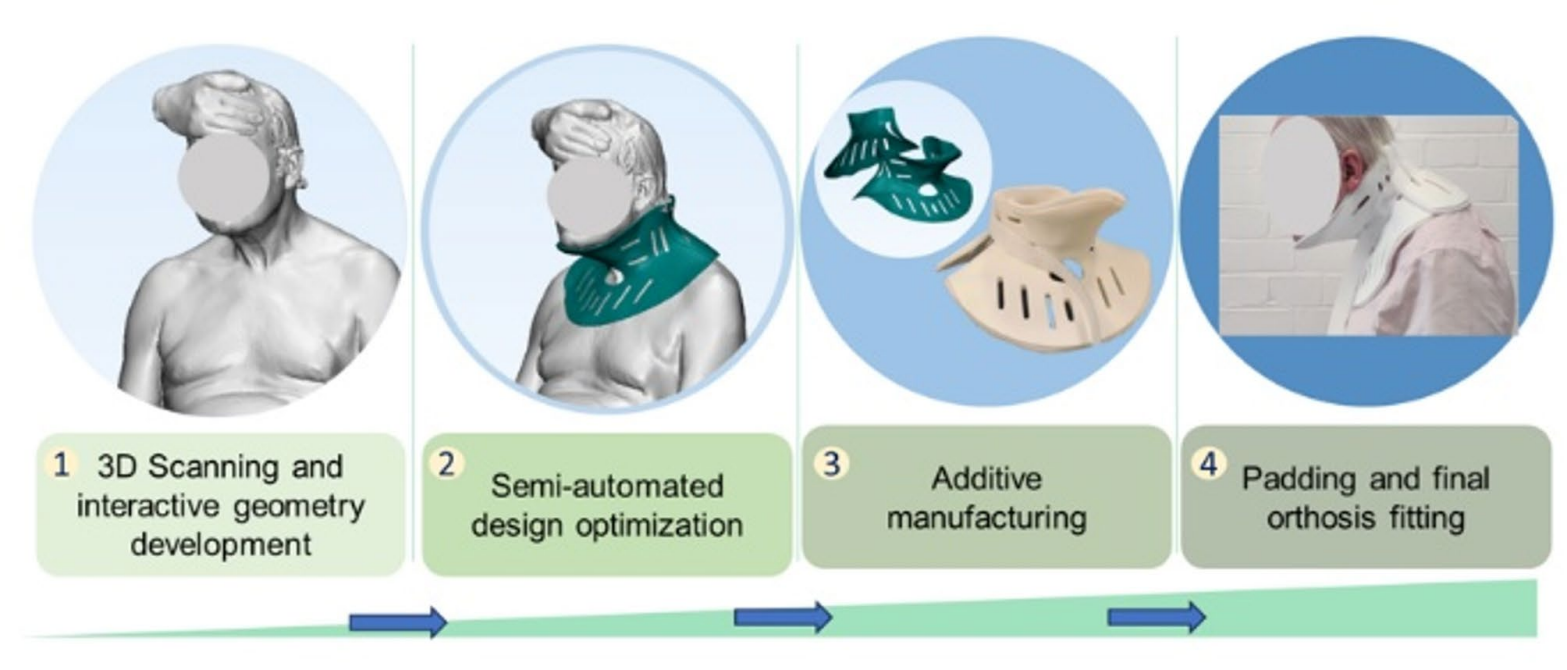
Schematic presentation of the workflow for development of a bespoke cervical orthotic device (neck collar) adopted and modified from Hale et al. [17]

### Study design

Patients with DHS were enrolled by the clinical team at the Spinal Surgery Unit of a Commissioning Hospital in the UK. Using an optical scanner (Artec EVA, ARTEC 3D, Luxembourg), the head and neck areas of the patients were scanned while seated, with manual positioning of head and neck alignment by a medical specialist. Subsequently, the engineering team designed bespoke orthotic devices based on the scans. Before fitting the collar, each device was validated by senior orthotists from the Orthotic Unit of the Commissioning Hospital in the UK. The study followed a previously published protocol, encompassing scanning to device design and manufacturing processes (see Fig. 1) [17].

### Patient selection

Among the total 6 participants, 67% were female (F) candidates, while the remaining 33% were male (M) candidates, resulting in a male-to-female ratio of 1:2. The distribution of patients by age was as follows: 2 patients aged 60–65 years, 2 patients aged 65–75 years, and 2 patients aged 75–85 years. Majority of the patients were living with neurological and spinal disorders such as Parkinson’s disease or other conditions associated with DHS.

### Study planning

The protocol for this feasibility study was approved by the Royal National Orthopaedics Hospital- Research Ethics Committee (REC) under compassionate patient access through Surgical Innovation and New Techniques and Technology committee. The patient recruitment and selection process were supervised by a consultant neurosurgeon, while patient appointments and communication were managed by a research nurse. All methods were performed in accordance with the relevant guidelines and regulations. All participants provided written informed consent for data collection and processing for publication. Patient scanning took place at the orthotics centre in the presence of the clinical, engineering, and orthotics teams, resulting in the creation of bespoke 3D scans approved by the clinical team. These scans were then utilized by the engineering team to design, and 3D print the collar using PBF, with design approval obtained from the orthotics team prior to printing.

Selection and preparation of collar cushioning, as well as the fitting process, were carried out by the orthotics team. The final fitting of the collar was conducted jointly by the orthotics and clinical teams. Follow-up appointments were coordinated by the research nurse at the Commissioning Hospital in the UK. Data analysis and reporting were conducted collaboratively by the entire team involved in the study.

**Fig. 2:**
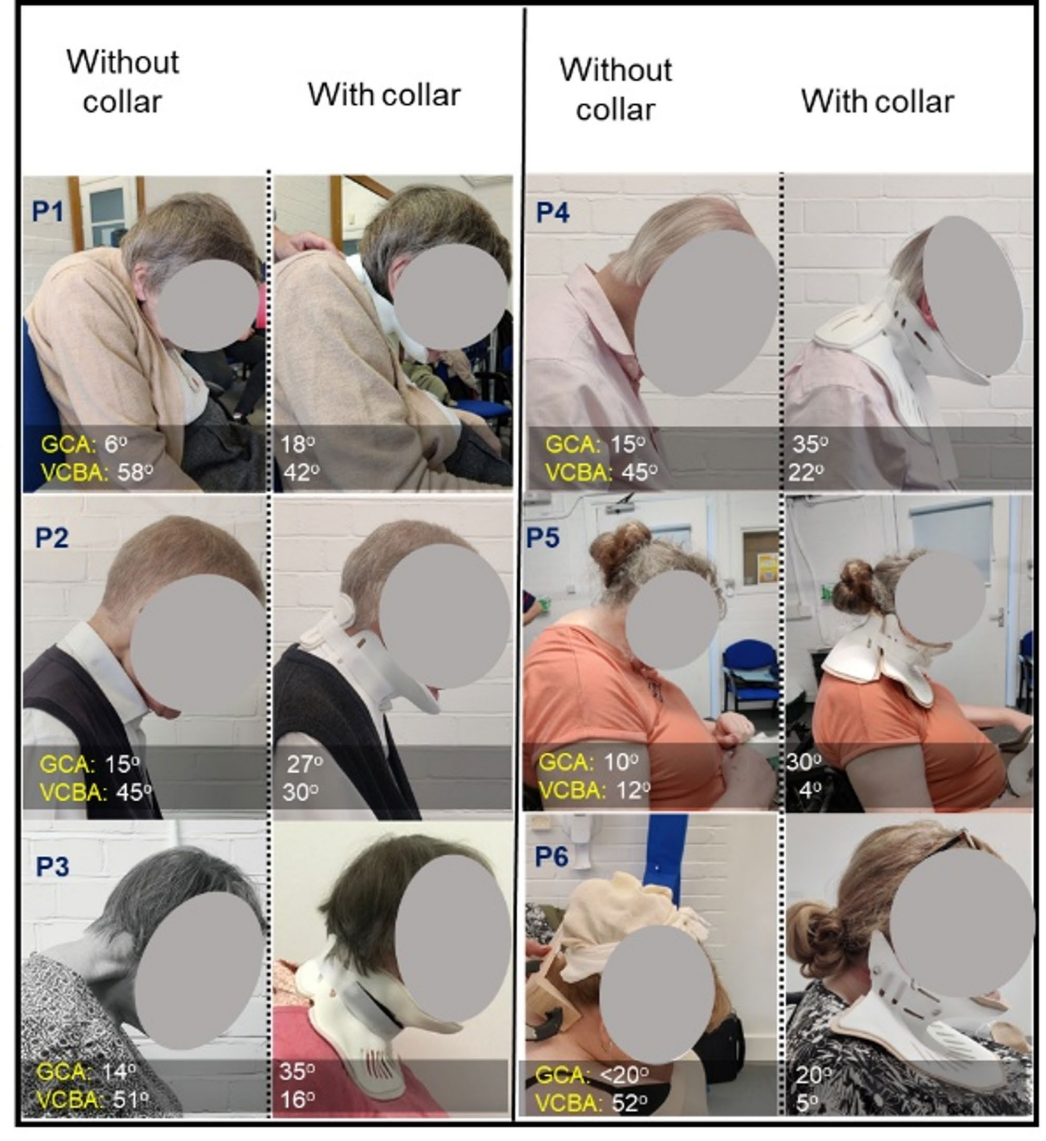
Representative photographs of the sagittal side view of the patients suffering from DHS, used to calculate GCA- Global Cervical Angle, VCBA- Vertical Chin Brow Angles using available clinical protocols [18–21], with and without collar used (*N* = 6). P 1-6- stands for patient number

### Design and 3D printing of collars

This study employed advanced 3D scanning and calibration techniques to design a tailored orthosis for each patient. Using the Artec EVA handheld scanner and Artec Studio software, a precise 3D surface scan of the patient’s head and neck was obtained. To ensure accuracy, patient landmark measurements were compared against global coordinates within the scan data.

The design phase commenced by importing the 3D scan mesh into Rhinoceros software, where a base 2D surface mesh was generated to match the patient’s unique anatomy. Notably, adjustments to the 2D form were minimal, as the scan already reflected the corrected head and neck position. Iterative modifications were made to optimize comfort, incorporating a 6 mm gap between the patient’s skin and the orthosis inner surface to accommodate padding and enhance breathability. Prototypes of the orthosis were then 3D printed via 3DPRINTUK PVT. LTD., UK (3D printing bureau) using EOS Formiga P110/P100 Printer, EOS GmbH, Germany) with Nylon-12 material. Final adjustments, including the addition of velcro strap closures and customized padding (already approved for orthotic use) using 6 mm closed-cell foam, were executed by the attending Orthotist prior to the fitting appointment.

Following fitting, patients were encouraged to evaluate the collar in their daily routines, providing feedback through a structured questionnaire designed to assess changes in clinical parameters and overall quality of life (please see Appendix A). This comprehensive approach ensured both precision in design and efficacy in function, tailored to each patient’s specific needs and preferences.

### Evaluation of collar performance

Neck collar performance was evaluated using various questionaries (please refer to Appendix A). Design based score (comfort, appearance, hygiene, and ease-of-putting on-and-off for the designed collars) was aimed to capture user requirements and data for bespoke collar design. Global Cervical Angle (GCA) and Vertical Chin Brow Angle (VCBA) were calculated from the sagittal photographs of the patients without and with-collar following the protocol from available clinical literature [18–21]. Neck Pain Visual Analog Score (neck pain intensity) and utility hours (wearing of the collars throughout a working day), were used to evaluate efficacy of collars in supporting patients during various activities and reducing neck pain.

## Results

### Global cervical angles (GCA) and vertical chin brow angles (VCBA)

**Fig. 3:**
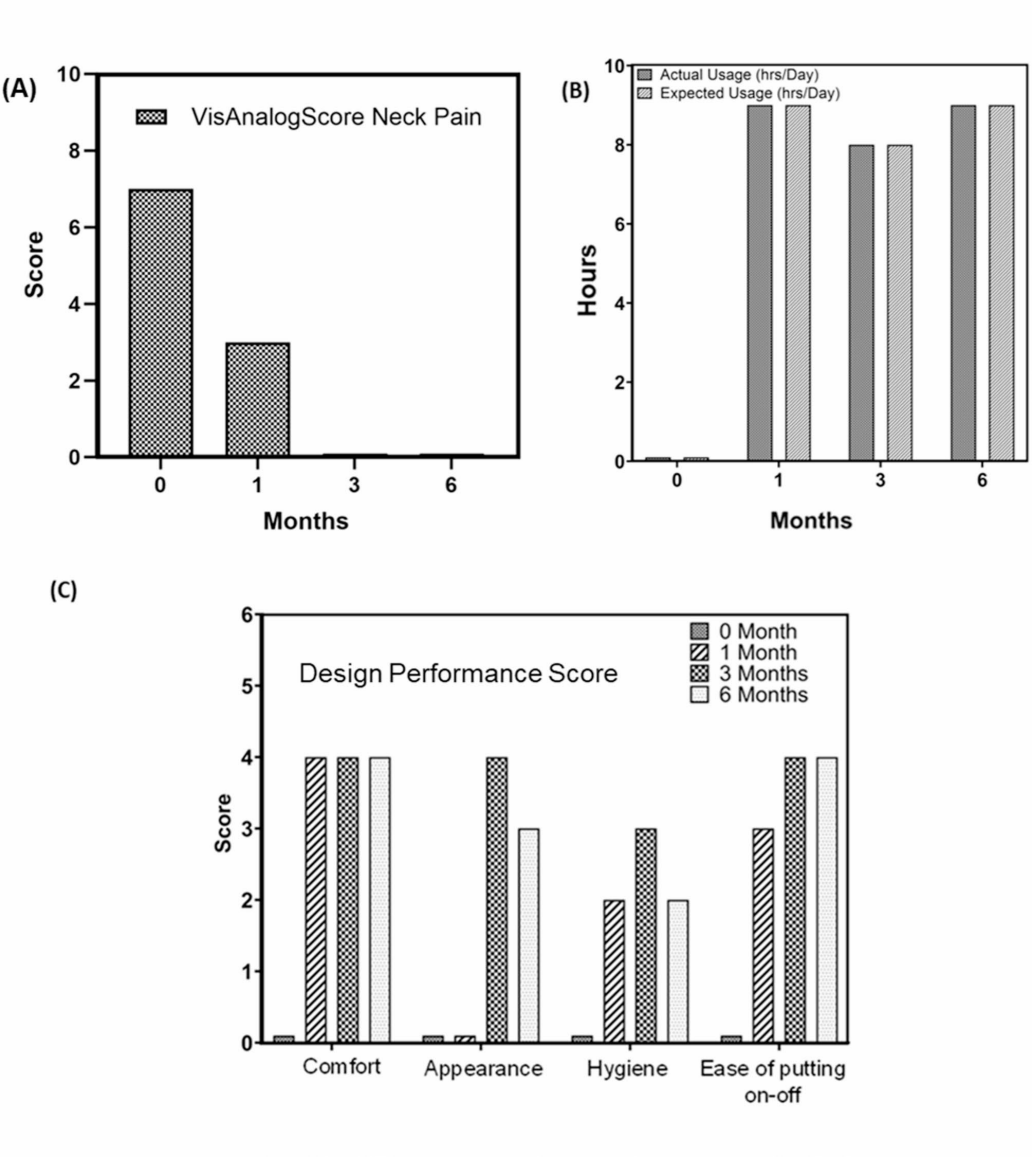
Collar performance analysis from feasibility trial for patient 3, provided with and without bespoke 3D printed Neck collar: (**A**) Neck Pain Visual Analog score, (scale 0–10) (**B**) Wearing collar hours analysis, and (**C**) Design performance score; (scale 0–5)

**Fig. 4:**
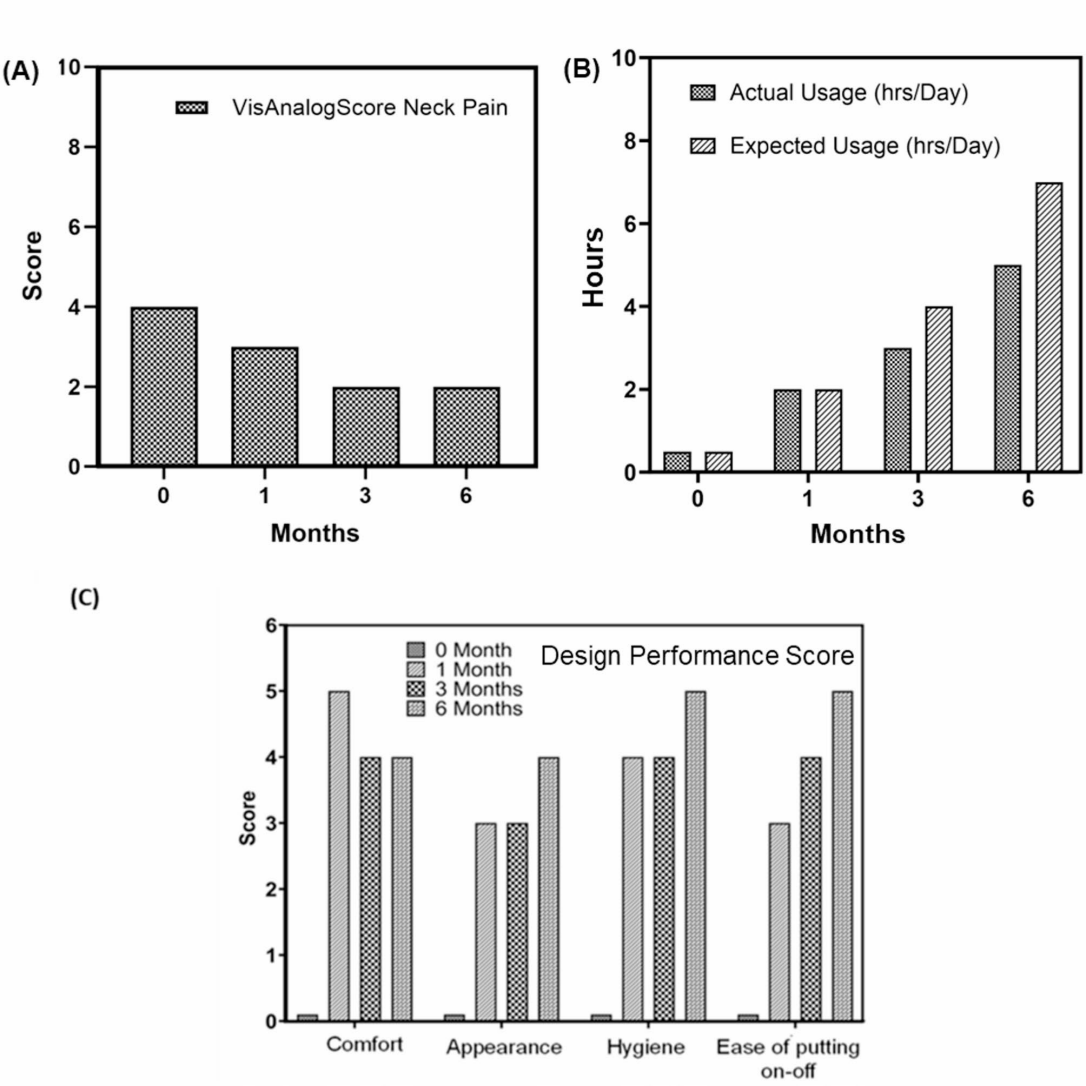
Collar performance analysis from feasibility trial for patient 4, provided with and without bespoke 3D printed Neck collar: (**A**) Neck Pain Visual Analog score (scale 0–10), (**B**) Wearing collar hours analysis, and (**C**) Design performance score; (scale 0–5)

Six patients were initially included in the study and provided with personalised neck collars (Fig. 2). Data analyses were performed using design-based scores and validated clinical scores, as depicted in Figs. 3 and 4.

Global cervical angles (GCA) and Vertical Chin Brow angles (VCBA) [18–21] were calculated from sagittal side view photographs of the patients (Fig. 2). Remarkably, the corrected cervical angles fell within the reported normal range (20–40 degrees) [22]. Moreover, visible enhancements in VCBA were observed following collar fitting, indicating improved straight visual gaze.

In all patients, there was a noticeable increasing trend in GCA, suggesting a positive impact of the neck collar on improving head and chin positions. Conversely, a decreasing trend in VCBA was noted in patients with collars as compared to their initial position without collars, affirming the efficacy of the personalized device in maintaining a straight gaze.

### Visual analogue score for neck pain, utility analysis, and design performance score

The first two patients (P1 & P2) underwent two collar revisions initially. However, unforeseen health complications prevented long-term follow-up, leading to their exclusion from the study due to inability to attend regular follow up sessions. Patients 3 and 4 were assessed at regular intervals for 6 months with their respective data presented in Figs. 3 and 4, highlighting observed progress over time.

For patients 5 and 6, the morphometric dimensions of their collars exceeded standard 3D printer capabilities due to larger anatomical shapes. This resulted in manufacturing delays and delayed delivery of the first prototypes. Although initial prototypes were provided at subsequent clinic visits, both patients withdrew from study for reasons not known and thus prevented from further data collection. Therefore, long-term follow-up data was only available and analysed for patients 3 and 4.

Figures 3 and 4 present a detailed quantitative analysis of the bespoke collars designed for patient 3 and 4, utilizing three parametric scores: Visual Analog Score for Neck Pain [23], utility analysis, and design performance score over a period of 6 months. Figures 3A and 4A demonstrate a reduction in neck pain following the use of patient-specific collars. These scores are unique to each patient. However, they showed time dependent improvement as patients got accustomed to neck collar device. Additionally, Figs. 3B and 4B reveal an increase in the hourly usage of the bespoke collars over time, correlating with observations of improved visual gaze positions. Possible theories of reduced fatigue and muscular strengthening are speculated from these findings. Assessment of collar performance from a design perspective is illustrated in Figs. 3C and 4C, showing improvement in comfort, appearance, and ease of use indices over time.

## Discussion

The study highlights the positive impact of bespoke collars on neck pain management and their potential to address unmet clinical need for DHS patients. During follow-up periods at 1 month, 3 months, and 6 months post-collar usage, patients reported improved ability to maintain a straight gaze position of the head and chin. Measurements of cervical angle and vertical chin brow angle (Fig. 2) demonstrated significant enhancement in visual gazing capability after collar usage, confirming the effectiveness of the bespoke device. In terms of neck pain management, for patient 3, at the end of sixth month, it was reported to be almost pain-free condition while using the collar for 8 h per day. In case of patient 4, similar trend was observed. However, larger morphometric dimensions for patients 5 and 6 posed manufacturing challenges, leading to delays in prototype delivery and subsequent modifications.

This research highlights the benefits of a digital workflow for creating custom cervical collars using 3D printing. This reliable process takes an average of 5 min for scanning, 24 h for design, and 72 h for 3D printing with PBF. Additional fitting and padding add about 3 h, bringing the total time to patient delivery to 3 days. While a cost comparison with commercially available collars was not possible, this digital method significantly reduces the time from scan to patient use compared to traditional casting and moulding techniques, which is both uncomfortable for patients and takes significantly longer time.

The accuracy and repeatability of the workflow are evident in the close agreement between design and final product, with positive user feedback on design and pain reduction. This digital approach using a PBF based 3D printing offers a faster and potentially more effective way to deliver customized orthotic care [24].

This small study (6 patients) with drop rate of 50%, explored the use of custom-made collars for neck pain relief and improved daily activities in DHS patients. While the limited sample size restricts broad conclusions, the results suggest these collars hold promise. The study also highlights the importance of accurate initial scans and collaboration with orthotists during the design process for optimal outcomes. Especially, during collar fitting for patients 5 and 6, it was noted that achieving a good initial corrected head position was challenging, emphasizing the importance of cautious initial scans conducted in the presence of both clinical and orthotics teams. The study acknowledges that for best practice, orthotists should be involved in applying rectification to the scans to optimize comfort and effectiveness of the final collars.

Furthermore, consistent collar use might promote sustained unsupported head positioning, which was reported by two patients, potentially leading to improved active head control during specific activities. This approach suggests the collars could act as proactive tools to strengthen the head and neck musculature, enhancing quality of life. Despite these promising findings, limitations include the small sample size, lack of data on patient variability, and no long-term follow-up beyond 1 year. However, the study successfully established a workflow for providing cervical collars to DHS patients, with positive patient-reported outcomes.

## Conclusions

This clinical feasibility study offers evidence supporting the positive impact of bespoke collars on lives of patients’ suffering from DHS. Notably, improvements in overall lifestyle, daily activities, and neck pain management were observed among individuals using customized neck collars. The tailored design and personalized functionality of the collars significantly contributed to enhancing users’ quality of life, marking a significant advancement in rehabilitative and supportive healthcare interventions.

These outcomes, indicative of an improved quality of life, highlight the multifaceted benefits of bespoke collars, positioning them as a promising option in orthotic care for patients with head and neck issues. However, further comprehensive investigations are necessary to validate and expand upon these preliminary findings. Such studies will provide a deeper understanding of the broader implications and potential applications of bespoke collars in the current healthcare landscape, benefiting the well-being of patients.

## Electronic supplementary material

Below is the link to the electronic supplementary material.

Supplementary Material 1

## Data Availability

Data is provided within the manuscript and supplementary information file. Some patient related data (identifiable) is not shared due to confidentiality.

## Abbreviations

3D: Three dimensional
PBF: Powder Bed Fusion
DHS: Dropped Head Syndrome
GCA: Global Cervical Angles
VCBA: Vertical Chin Brow Angles
