# Supplementary material for: Clinical case study on custom 3D printed collars for dropped head syndrome patients
Source: 3D Print Med. 2025 Jun 5;11:23. doi: 10.1186/s41205-025-00274-x (PMC12139371; doi:10.1186/s41205-025-00274-x)
Supplement: Supplementary file 1 — Supplementary Material 1 [file 41205_2025_274_MOESM1_ESM.pdf]

Neck Disability Index

Please complete this questionnaire. It is designed to give us information as to how your neck (or arm) trouble affects your ability to manage in everyday life. Please answer **every section**. Tick **one box only** in each sec ion that mos closely describes you **today**.

1. Pain Intensity

- ☐ I have no pain at the moment
- ☐ The pain is very mild at the moment
- ☐ The pain is moderate at the moment
- ☐ The pain is fairly severe at the moment
- ☐ The pain is very severe at the moment
- ☐ The pain is the worst imaginable at the moment

2. Personal care (washing, dressing etc)

- ☐ I can look after myself normally without causing extra pain
- ☐ I can look after myself normally but it is very painful
- ☐ It is painful to look after myself and I am slow and careful
- ☐ I need some help but manage most of my personal care
- ☐ I need help every day in most aspects of self-care
- ☐ I do not get dressed, wash with difficulty and stay in bed

3. Lifting

- ☐ I can lift heavy weights without extra pain
- ☐ I can lift heavy weights but it gives extra pain
- ☐ Pain prevents me from lifting weights off the floor but I can manage if they are conveniently positioned, eg on a table
- ☐ Pain prevents me from lifting weights off the floor but I can manage light to medium weights if they are conveniently positioned
- ☐ I can lift only very light weights
- ☐ I cannot lift or carry anything at all

4. Reading

- ☐ I can read as much as I want to with no pain in my neck
- ☐ I can read as much as I want to with slight pain in my neck
- ☐ I can read as much as I want to with moderate pain in my neck
- ☐ I cannot read as much as I want because of moderate pain in my neck
- ☐ I can hardly read at all because of severe pain in my neck
- ☐ I cannot read at all

5. Headaches

- ☐ I have no headaches at all
- ☐ I have slight headaches which come infrequently
- ☐ I have moderate headaches which come infrequently
- ☐ I have moderate headaches which come frequently
- ☐ I have severe headaches which come frequently
- ☐ I have headaches almost all the time

6. Concentration

- ☐ I can concentrate fully when I want to wi h no difficulty
- ☐ I can concentrate fully when I want to wi h slight difficulty
- ☐ I have a fair degree of difficul y in concentra ing when I want to
- ☐ I have a lot of difficulty concentrating when I wan to
- ☐ I have a great deal of difficulty concentrating when I want to
- ☐ I cannot concentrate at all

7. Work

- ☐ I can do as much work as I want to
- ☐ I can only do my usual work, but no more
- ☐ I can do most of my usual work, but no more
- ☐ I cannot do my usual work
- ☐ I can hardly do any work at all
- ☐ I cannot do any work at all

8. Driving

- ☐ I can drive my car without any neck pain
- ☐ I can drive my car as long as I want wit sligh pain in my neck
- ☐ I can drive my car as long as I want with moderate pain in my neck
- ☐ I cannot drive my car as long as I want because of moderate pain in my neck
- ☐ I can hardly drive at all because of severe pain in my neck
- ☐ I cannot drive my car at all

9. Sleeping

- ☐ I have no trouble sleeping
- ☐ My sleep is slightly disturbed (less than 1 hour sleepless)
- ☐ My sleep is mildly disturbed (1-2 hours sleepless)
- ☐ My sleep is moderately disturbed (2-3 hours sleepless)
- ☐ My sleep is greatly disturbed (3-5 hours sleepless)
- ☐ My sleep is completely disturbed (5-7 hours)

10. Recreation

- ☐ I am able to engage in all of my recreational activities with no neck pain at all
- ☐ I am able to engage in all of m recreational activities with some pain in my neck
- ☐ I am able to engage in most, but not all o m recreational activities because of pain in m nec
- ☐ I am able to engage in a few of m recreational activities because of pain in m nec
- ☐ I can hardly do any recreational acti itie becau e of pain in my neck
- ☐ I cannot do any recreational acti itie at all

Name: ____

Date of Birth: ____/____/______

Date: ___/___/_____

Visual Analogue Score: Neck and Arm Pain

1. Please mark on the line below how much pain you have had from your neck, on average, over the past week:

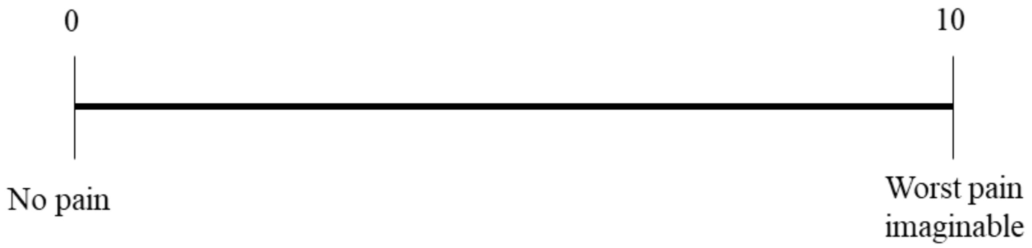

2. Please mark on the line below how much pain you have had from your worst arm, on average, over the past week:

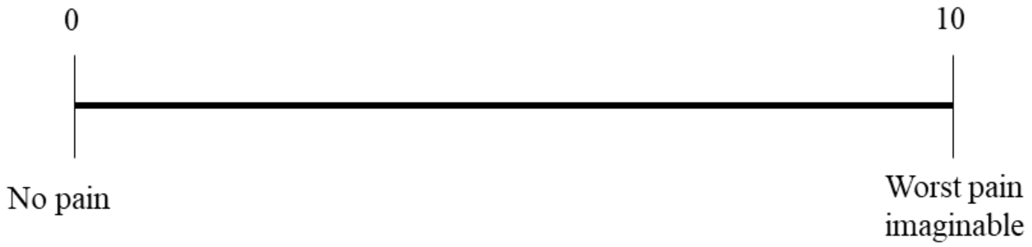

3. If you have pain in other arm, please mark on the line below how much pain you have had on average, over the past week:

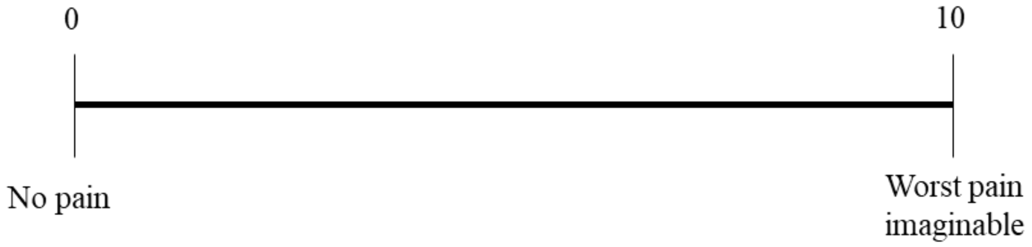

Neck Support Questionnaire: First Visit

Patient ID:

Date:

Primary Diagnosis of the patient:

This questionnaire is designed to assess neck collars in people living with neck disorders.

1. Please state how long you typically wear a neck collar a day?	1 2 3 4 5 6 7 8 9 10<br>I don't use for long time as its too uncomfortable	
2. How long do you think you need to wear a collar?	1 2 3 4 5 6 7 8 9 10<br>I don't use for long time as its too uncomfortable	
3. What activities would a collar help with?<br>4. Which activities does your current collar actually help with?	Eating ☐<br>Watching TV ☐<br>Travelling (i.e in a car) ☐<br>Bathing/Showering ☐<br>Clothing/getting ready ☐<br>Personal care (Makeup shaving)☐<br>Around the house ☐<br>In the Kitchen ☐<br>Transferring from one place to another ☐<br>Other: Click or tap here to enter text.	
5. Do you think a collar made specifically for you would help?	Yes ☐	No ☐
a. Based on a current collar usage rate the following measures<br>(5 = best)	Comfort<br>1 2 3 4 5<br>Appearance<br>1 2 3 4 5<br>Hygiene<br>1 2 3 4 5<br>Ease of Use<br>1 2 3 4 5<br>Ease of putting On & Off<br>1 2 3 4 5	

b. What are the top three priorities in order of preference:<br>(Please tick)	Comfort<br>Appearance<br>Hygiene<br>Ease of use<br>Ease of putting on and off

6. How many collars did you try on before you found one that fitted?

1 2 3 4 5 6 7 8 9 10<br>I have not tried any

7. How many collars did you try longer than a day?

1 2 3 4 5 6 7 8 9 10<br>I have not tried any

8. How did the collar fit, any areas of discomfort? Please refer to the diagram below when indicating to areas of discomfort

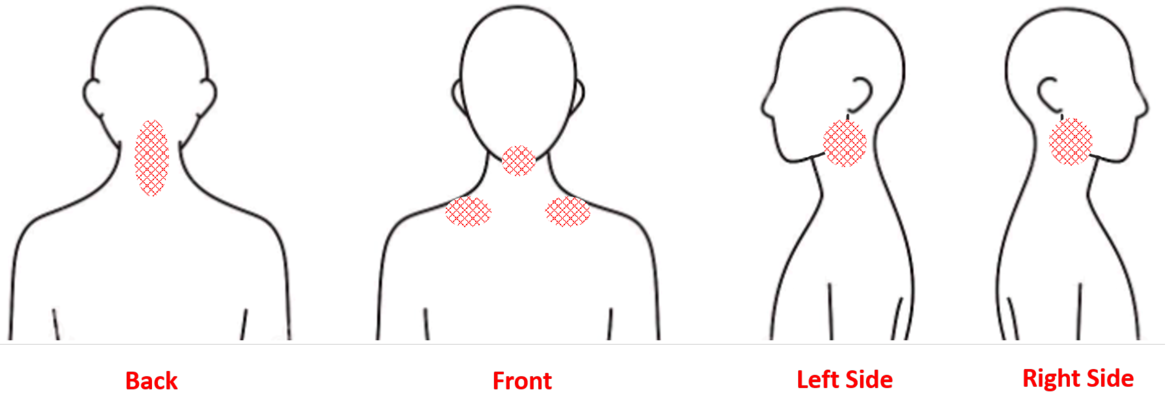

***Interactive map – click on the hatches**

Neck Support Questionnaire

Please refer to the following statements and place a mark on the circle you feel best describes your experience regarding previous neck collars, as shown in the example below.

The collars cause no restriction to my natural breathing ability

Strongly agree	Agree	Agree somewhat	Neither agree nor disagree	Disagree somewhat	Disagree	Disagree strongly
☐	☐	☐	☐	☐	☒	☐

The collars support my head effectively

Strongly agree	Agree	Agree somewhat	Neither agree nor disagree	Disagree somewhat	Disagree	Disagree strongly
☐	☐	☐	☐	☐	☐	☐

The collars allow natural movement of my head.

Strongly agree	Agree	Agree somewhat	Neither agree nor disagree	Disagree somewhat	Disagree	Disagree strongly
☐	☐	☐	☐	☐	☐	☐

I am able to fit the collars on my own

Strongly agree	Agree	Agree somewhat	Neither agree nor disagree	Disagree somewhat	Disagree	Disagree strongly
☐	☐	☐	☐	☐	☐	☐

The collars cause no restriction to my natural breathing ability

Strongly agree	Agree	Agree somewhat	Neither agree nor disagree	Disagree somewhat	Disagree	Disagree strongly
☐	☐	☐	☐	☐	☐	☐

I experience no additional difficulties eating a meal when wearing the collar

Strongly agree	Agree	Agree somewhat	Neither agree nor disagree	Disagree somewhat	Disagree	Disagree strongly
☐	☐	☐	☐	☐	☐	☐

Neck Support Questionnaire

I experience no additional problems drinking when wearing the collar

Strongly agree	Agree	Agree somewhat	Neither agree nor disagree	Disagree somewhat	Disagree	Disagree strongly
☐	☐	☐	☐	☐	☐	☐

The collar causes no restriction to my natural swallowing ability

Strongly agree	Agree	Agree somewhat	Neither agree nor disagree	Disagree somewhat	Disagree	Disagree strongly
☐	☐	☐	☐	☐	☐	☐

Neck Support Questionnaire

Please tick the collar you primarily use:

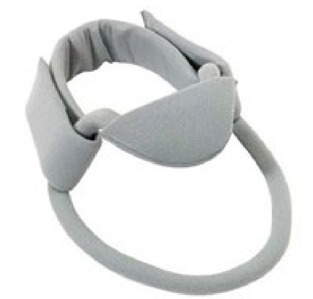	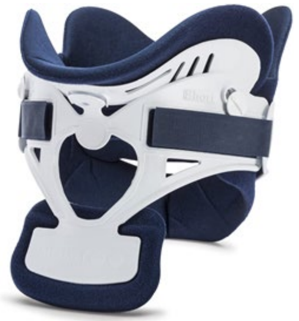	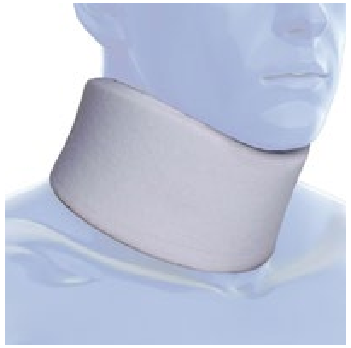	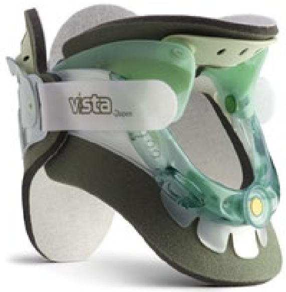	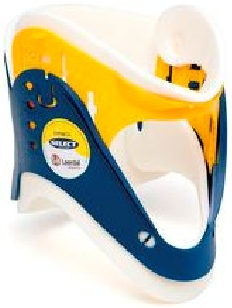
☐	☐	☐	☐	☐
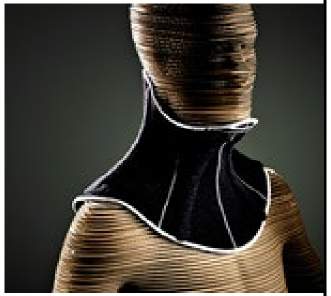	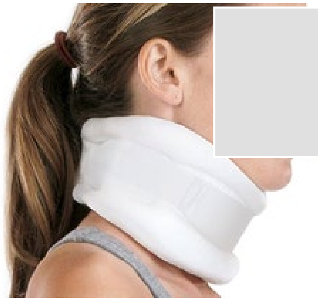	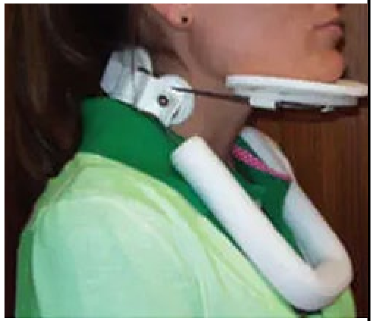	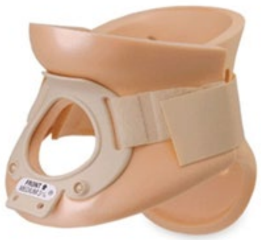	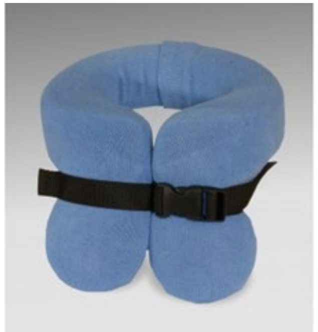
☐	☐	☐	☐	☐

Neck Support Questionnaire

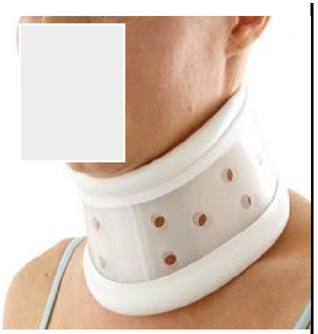	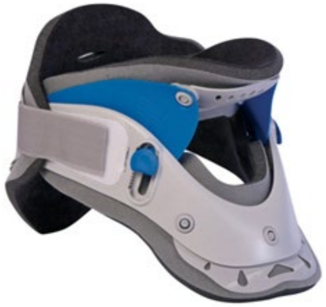			
☐	☐			

Neck Support Questionnaire: Post use Prototype 1

Patient ID:

Date:

Primary Diagnosis of the patient:

This questionnaire is designed to assess your current bespoke neck collars

1. Please state how long you typically wore a neck collar a day?	1 2 3 4 5 6 7 8 9 10 hours<br><br>I don't use for long time as its too uncomfortable	
2. How long do you think you need to wear a collar?	1 2 3 4 5 6 7 8 9 10 hours<br><br>I don't use for long time as its too uncomfortable	
3. What activities are you able to perform with the current collar?	Eating ☐<br>Watching TV ☐<br>Travelling (i.e in a car) ☐<br>Bathing/Showering ☐<br>Clothing/getting ready ☐<br>Personal care (Makeup shaving)☐<br>Around the house ☐<br>In the Kitchen ☐<br>Transferring from one place to another ☐<br>Other:	
4. Do you think the specifically made-for-you collar helped?	Yes ☐	No ☐
a. Based on a current collar usage rate the following measures<br><br>(5 = best)	Comfort<br>1 2 3 4 5<br>Appearance<br>1 2 3 4 5<br>Hygiene<br>1 2 3 4 5<br>Ease of Use<br>1 2 3 4 5<br>Ease of putting On & Off<br>1 2 3 4 5	

b. What are the top three priorities in order of preference the current collar achieved:<br>(Please tick)	Comfort<br>Appearance<br>Hygiene<br>Ease of use<br>Ease of putting on and off

5. How did the collar fit, any areas of discomfort? Please refer to the diagram below when indicating to areas of discomfort

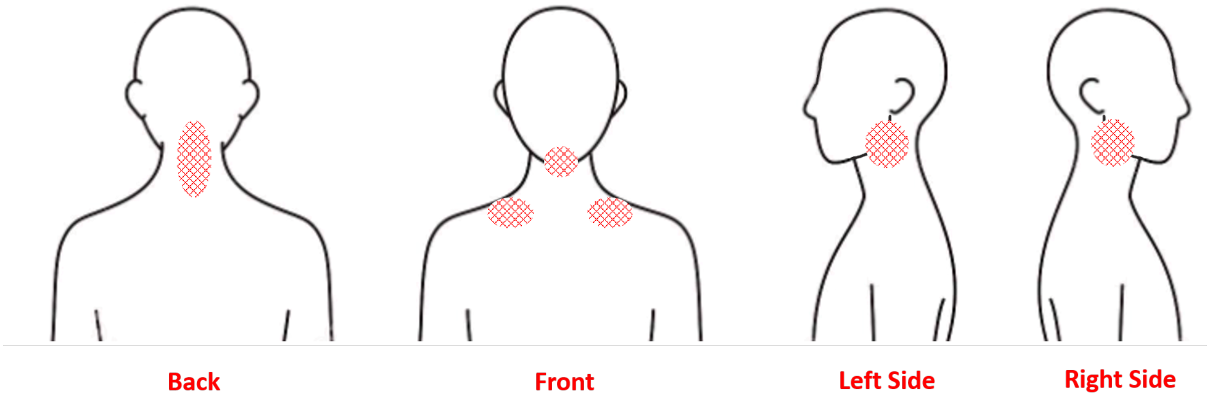

***Interactive map – circle area which describe area of discomfort**

Neck Support Questionnaire: Post use Prototype 1

Please refer to the following statements and place a mark on the circle you feel best describes your experience regarding neck collar, as shown in the example below.

The collars cause no restriction to my natural breathing ability

Strongly agree	Agree	Agree somewhat	Neither agree nor disagree	Disagree somewhat	Disagree	Disagree strongly
☐	☐	☐	☐	☐	☒	☐

The collar supports my head effectively

Strongly agree	Agree	Agree somewhat	Neither agree nor disagree	Disagree somewhat	Disagree	Disagree strongly
☐	☐	☐	☐	☐	☐	☐

The collar allows natural movement of my head.

Strongly agree	Agree	Agree somewhat	Neither agree nor disagree	Disagree somewhat	Disagree	Disagree strongly
☐	☐	☐	☐	☐	☐	☐

I am able to fit the collar on my own

Strongly agree	Agree	Agree somewhat	Neither agree nor disagree	Disagree somewhat	Disagree	Disagree strongly
☐	☐	☐	☐	☐	☐	☐

The collar causes no restriction to my natural breathing ability

Strongly agree	Agree	Agree somewhat	Neither agree nor disagree	Disagree somewhat	Disagree	Disagree strongly
☐	☐	☐	☐	☐	☐	☐

I experience no additional difficulties eating a meal when wearing the collar

Strongly agree	Agree	Agree somewhat	Neither agree nor disagree	Disagree somewhat	Disagree	Disagree strongly
☐	☐	☐	☐	☐	☐	☐

Neck Support Questionnaire: Post use Prototype 1

I experience no additional problems drinking when wearing the collar

Strongly agree	Agree	Agree somewhat	Neither agree nor disagree	Disagree somewhat	Disagree	Disagree strongly
☐	☐	☐	☐	☐	☐	☐

The collar causes no restriction to my natural swallowing ability

Strongly agree	Agree	Agree somewhat	Neither agree nor disagree	Disagree somewhat	Disagree	Disagree strongly
☐	☐	☐	☐	☐	☐	☐

Please feel free to write any other comments or observations you have regarding your experience of wearing the collar.
